# Investigating possible ethnicity and sex bias in clinical examiners: an analysis of data from the MRCP(UK) PACES and nPACES examinations

**DOI:** 10.1186/1472-6920-13-103

**Published:** 2013-07-30

**Authors:** I C McManus, Andrew T Elder, Jane Dacre

**Affiliations:** 1 Academic Centre for Medical Education, Division of Medical Education, University College London, Gower Street, London WC1E 6BT, UK; 2 Research Department of Clinical, Educational and Health Psychology, Division of Psychology and Language Sciences, University College London, Gower Street, London WC1E 6BT, UK; 3 Examinations Department, MRCP(UK) Central Office, 11 St. Andrews Place, Regent’s Park, London NW1 4LE, UK; 4 College of Medicine and Veterinary Medicine, The University of Edinburgh, The Queen’s Medical Research Institute, 47 Little, Crescent, Edinburgh EH16 4TJ, France

**Keywords:** Examiner bias, Hawks and doves, Sex, Ethnicity

## Abstract

**Background:**

Bias of clinical examiners against some types of candidate, based on characteristics such as sex or ethnicity, would represent a threat to the validity of an examination, since sex or ethnicity are ‘construct-irrelevant’ characteristics. In this paper we report a novel method for assessing sex and ethnic bias in over 2000 examiners who had taken part in the PACES and nPACES (new PACES) examinations of the MRCP(UK).

**Method:**

PACES and nPACES are clinical skills examinations that have two examiners at each station who mark candidates independently. Differences between examiners cannot be due to differences in performance of a candidate because that is the same for the two examiners, and hence may result from bias or unreliability on the part of the examiners. By comparing each examiner against a ‘basket’ of all of their co-examiners, it is possible to identify examiners whose behaviour is anomalous. The method assessed hawkishness-doveishness, sex bias, ethnic bias and, as a control condition to assess the statistical method, ‘even-number bias’ (i.e. treating candidates with odd and even exam numbers differently). Significance levels were Bonferroni corrected because of the large number of examiners being considered.

**Results:**

The results of 26 diets of PACES and six diets of nPACES were examined statistically to assess the extent of hawkishness, as well as sex bias and ethnicity bias in individual examiners. The control (odd-number) condition suggested that about 5% of examiners were significant at an (uncorrected) 5% level, and that the method therefore worked as expected. As in a previous study (*BMC Medical Education*, 2006, 6:42), some examiners were hawkish or doveish relative to their peers. No examiners showed significant sex bias, and only a single examiner showed evidence consistent with ethnic bias. A re-analysis of the data considering only one examiner per station, as would be the case for many clinical examinations, showed that analysis with a single examiner runs a serious risk of false positive identifications probably due to differences in case-mix and content-specificity.

**Conclusions:**

In examinations where there are two independent examiners at a station, our method can assess the extent of bias against candidates with particular characteristics. The method would be far less sensitive in examinations with only a single examiner per station as examiner variance would be confounded with candidate performance variance. The method however works well when there is more than one examiner at a station and in the case of the current MRCP(UK) clinical examination, nPACES, found possible sex bias in no examiners and possible ethnic bias in only one.

## Background

Bias in any examination is a threat to its validity, marks awarded being dependent not only upon a candidate’s performance, but also upon other factors which generally are called ‘construct-irrelevant’. In clinical examinations, where a candidate’s performance is observed by an examiner, there is a potential risk that an examiner’s judgements depend in part upon the examiner’s personality, attitudes or predispositions in general (resulting most obviously in being a ‘hawk’ or a ‘dove’). A further possibility is that the personal characteristics of the candidate, most obviously their sex or ethnicity, may modify an examiner’s response to the candidate’s performance, a situation which can be conceptualised as being, say, ‘hawkish’ to one sex and ‘doveish’ to the other.

Although most clinical examiners are aware of the possibility of there being ‘hawks’ and ‘doves’ [1], formal statistical analyses to identify the behaviour are rare [2]. In a previous study [3] we used multi-facet Rasch analysis, using the program *Facets*, to assess whether examiners on the MRCP(UK) PACES examination were hawks or doves, and found that there was good statistical evidence that some examiners were indeed more hawkish or doveish than other examiners, but the overall impact upon final outcome was small, not least because each candidate was assessed by ten different examiners, diluting the effect of any individual hawk or dove. Although *Facets* does provide a way of approaching the hawk-dove problem, it is much more complex to use to assess the interaction of examiner identity and candidate sex or ethnicity upon outcome. Analyses of possible bias are also complicated by the sex and ethnicity of candidates seeming to have a direct impact upon performance, not only in postgraduate examinations [4], but also in undergraduate examinations [5], with it being important to note that the differences apply to machine-marked examinations as well as clinical assessments. Any simple comparison of marks awarded by sex or ethnicity of candidates thereby runs the risk of detecting genuine candidate differences rather than genuine examiner differences. Although in the past we have examined the effect of examiner sex and ethnicity in relation to candidate sex and ethnicity [4], we have only done that on aggregated results, and not at the level of individual examiners. In this paper we describe a different, somewhat simpler, statistical approach to the problem of identifying individual examiners whose personal biases concerning sex or ethnicity may influence results, and at the same time we also assess the hawkishness or doveishness of examiners.

MRCP(UK) (Membership of the Royal Colleges of Physicians of the United Kingdom) involves three examinations, Part 1 and Part 2 being multiple-choice assessments of clinical knowledge, and PACES (Practical Assessment of Clinical Examination Skills) being an OSCE-style examination. The parts of MRCP(UK) are usually taken in order in the 2 to 4 years after graduation, making it in effect an ‘exit examination’ from core medical training, and an ‘entry examination’ for specialist training in internal medicine. PACES was introduced in 2001 to replace a traditional “long-case, short-case, viva” clinical examination, and comprised five stations, each of 20 minutes, in which each candidate was assessed by two senior clinical examiners marking independently [6]. A total of ten examiners therefore assessed each candidate.

The examination was modified in 2009 into the current “new” (n) PACES format [7,8]. In nPACES examiners continue to work in pairs across the five stations and each candidate is assessed by ten examiners. Three stations (Respiratory and Abdominal/Cardiovascular and Nervous System/Integrated clinical assessment involving two brief clinical consultations) included real patients and focussed on the examination and interpretation of actual physical signs, and two stations used simulated patients, one assessing history-taking skills and the other assessing communication skills in more difficult situations, often involving ethical issues. In the stations with simulated patients, two examiners were also present and it was they who marked the station. The nPACES content was broadly similar to that of the earlier PACES except that the Integrated Clinical Assessment station had replaced an earlier station that focussed mainly on physical examination skills alone. The assessment methodology and marking structure changed from what in PACES was known as “compensated encounter-based marking” to what in nPACES was “uncompensated skills-based marking”. In association with this change the total number of independent judgements made about each candidate by the ten examiners increased from 14 to 86, candidates being assessed on seven separate skills (although not all skills could be assessed at all stations).

All UK-based PACES/nPACES examiners are required to undergo ethnicity and diversity training and their status in that regard is monitored by MRCP(UK). Examiner pairings and assignment of examiners to each station are random and change within and across individual examining days. In a typical examining day three carousels of five candidates will be examined by a team of ten examiners. Examiners assess candidates in pairs but mark independently, having personally assessed the real or simulated patient or patients that their candidates will be seeing and agreed on marking criteria with their paired examiner in a process known as “calibration”.

PACES and nPACES are high stakes, postgraduate clinical skills assessments with many candidates who have qualified outside of the UK, the exam being held in the UK and nine international centres. A substantial proportion of candidates is non-white or female, but a majority of examiners are white and male. White candidates and female candidates have higher pass rates than non-white and male candidates [4]. Examination in international centres always have visiting examiners from the UK, and all candidates in international centres are seen by a UK and a local examiner at each station.

The PACES and nPACES assessments have an intrinsic advantage over many other clinical examinations for the sort of analysis we wish to carry out, in that at each encounter each candidate is always seen by two examiners, both of whom make their assessments independently. In that situation, if two examiners at any individual encounter differ in their judgements it could be for one of several reasons: a) one examiner may systematically award higher or lower marks than the other (the hawk-dove effect); b) one examiner could be more variable than the other (either in the sense of spreading their judgements more widely (greater variance), or in having more measurement error than the other (and in the extreme case they would be making random judgements); or c) one examiner could be treating some types of candidates differently to the other examiner (perhaps due to bias in terms of sex, ethnicity or other personal characteristics). Of course all of those effects could also be present in a more typical clinical examination in which there is only one examiner per encounter, but in that case the effects would also be confounded with candidate performance, and distinguishing examiner variance from candidate variance would be much less easy. With two examiners, the candidate variance within any particular encounter is constant, and therefore any differences must be due to examiner variance of some form. That is the essence of the analysis described here.

If data were available only for a single pair of examiners at a single encounter it would not be possible, when examiner A marks a candidate lower than does examiner B, to tell whether A is more hawkish or B more doveish on average. However in PACES and nPACES, each candidate is seen by ten separate examiners (working as five pairs). Examiners are paired with the same co-examiner for a particular ‘carousel’ of five candidates whom they see at their station, but then for other carousels they are paired with other examiners. The result is that moderately experienced examiners will have co-examined with a large number of other examiners. The PACES/nPACES regulations expect that an examiner will see at least 30 candidates a year (six carousels, typically over two days), and most examiners have examined for several years, meaning that over two years examiners will have co-examined with at least a dozen or more other examiners (see Results section for detailed statistics). While it is not possible to tell whether, say, examiner A is a dove or B is a hawk after considering just that pair, when A has co-examined with, say, 12 other examiners, C to N, and has made a series of judgements about the same candidates with each of those co-examiners, then if A is indeed a hawk then their marks should be consistently lower when compared with the average of all of their co-examiners. Each examiner is therefore being compared with the ‘basket’ of responses made by all of their co-examiners, and the question can therefore be asked of whether statistically that individual examiner’s judgements are significantly different from the aggregate of their co-examiners. Note that this method does not compare an examiner with *all* of the other examiners in general, but instead it compares the examiner precisely with all of the other examiners *with whom they have actually co-examined* (and therefore have seen the identical candidates performing identical tasks). That gives the method much greater statistical power, as well much greater face validity. Examiners may see different types of candidate, both in demographic terms, and, in particular, candidates of differing overall ability level (and candidate ability often correlates with other variables, such as time, date or place of examining) making it difficult to know in general whether an examiner gives lower scores because the candidates are less good, or because the examiner is more hawkish. With two examiners that explanation no longer becomes a possibility, and none of the analyses we report on two examiners can be secondary to differences in case-mix. The power and sensitivity of the method depend, inevitably, on the number of candidates an examiner has seen, and also to some extent depend on the number of their co-examiners.

MRCP(UK) has recorded self-declared ethnicity of its candidates and examiners for several years and has previously reported on the relative performance of candidates according to their ethnicity and gender [4]). The present analysis was prompted by two further factors. Firstly, the GMC has recently suggested that all UK Colleges and Faculties providing postgraduate medical assessments should investigate the possibility of ethnic and sex bias in their assessments. Secondly, MRCP(UK) receives very occasional complaints from candidates who believe that the assessment is, in some way, biased against them. There was therefore a desire to assess the extent to which examiners may be biased against candidates of a particular sex or ethnicity, to assess whether they may be hawks and doves overall, and to consider whether such analysis could be used to inform the investigation of an individual candidate’s allegations of bias by individual examiners. As there was an inevitable concern that any method may be unduly sensitive or may produce false positives, we therefore also chose to have a ‘control condition’, something which could reasonably be assumed to have no true impact on examiner or candidate behaviour, and for that we considered whether the RCP code number of the candidate was odd or even.

PACES and nPACES are fortunate in having two examiners at each station, allowing the analyses described above (and that undoubtedly contributes to the reliability of the examination [9]). Many other clinical examinations however have only a single examiner at each station. An important practical question concerns whether it is possible to detect examiner bias under such circumstances. The PACES data can be used to simulate the effects of having only a single examiner, by considering the marks of just one of the two examiners at a station. We do that in two ways, to be described below, and it will be seen that both have serious problems. That conclusion has implications for methods that claim to identify ethnic bias, sex bias or undue degrees of hawkishness or doveishness in exams with only a single examiner at each station.

## Methods

A statistical analysis was carried out of all candidates at all examination centres for the first 26 diets of PACES, the original form of the examination, held from 2001/1 to 2009/2, and for the next six diets of nPACES, diets 27–32, held from 2009/3 to 2011/2. In PACES the examiners at three of the five stations independently gave one mark on a four-point scale (Clear Fail, Fail, Pass, Clear Pass), and at two of the stations independently awarded two separate marks on the same four-point scales but for the assessment of two different patients. An individual examiner could therefore give either five or ten separate marks in each carousel of five candidates, according to the station on which they were examining. For nPACES, the two examiners at each of the five stations independently assessed between four and seven separate skills (described briefly as “Physical examination”, “Interpreting physical signs”, “Clinical communication”, “Differential Diagnosis”, “Clinical judgement”, “Managing patient concerns”, and “Maintaining patient welfare” [8]), the particular skills depending on the nature of the station, each skill being assessed on a three-point scale (Unsatisfactory, Borderline, Satisfactory). In this form of the examination an individual examiner in each carousel of five candidates could award between 20 and 35 separate marks, depending on the station on which they were examining.

Ethnicity in the MRCP(UK) database is reported in terms of 19 categories, modified to take into account the international pool of candidates taking the examination. Examples of the data grouped into seven superordinate categories (plus Unknown) are shown in Table 1 of a previous paper [4]. For present purposes, and as is conventional elsewhere [5,10], we grouped all non-white groups together and contrasted them with the white group. Since white and non-white groups are broadly equivalent in size that results in maximal power.

**Table 1:** The table shows separately for analyses of hawk-dove differences, male–female differences, White-nonWhite differences, and differences between odd and even numbered candidates (see columns), the numbers of examiners who reached statistical significance (rows) on various criteria

	**Hawk-Dove**		**Male–female**		**White-NonWhite**		**Odd-Even numbering**	
	**Positive: Examiner hawkish**		**Positive: Males score higher than females**		**Positive: Whites score higher than non-Whites**			
	**PACES**	**nPACES**	**PACES**	**nPACES**	**PACES**	**nPACES**	**PACES**	**nPACES**
Negative effect: P < .05 corrected	34 (1.9%)	35 (2.3%)	0	0	2 (0.1%)	1 (0.1%)	0	0
Negative effect: P < .05 uncorrected (chance expectation = 2.5%)	198 (11.1%)	235 (15.7%)	73 (4.1%)	63 (4.2%)	73 (4.4%)	48 (3.6%)	60 (3.2%)	51 (3.2%)
Not significant (uncorrected, p > .05)	1339 (74.8%)	989 (66.0%)	1638 (91.5%)	1379 (92.1%)	1491 (90.4%)	1229 (92.2%)	1680 (93.9%)	1396 (93.2%)
Positive effect: P < .05 uncorrected (chance expectation = 2.5%)	192 (10.7%)	200 (13.4%)	79 (4.4%)	55 (3.7%)	82 (5.0%)	55 (4.1%)	50 (3.0%)	51 (3.6%)
Positive effect: P < .05 corrected	27 (1.5%)	39 (2.6%)	0	0	1 (0.1%)	0	0	0
*N examiners*	*1790*	*1498*	*1790*	*1497*	*1649*	*1333*	*1790*	*1498*

Levels of statistical significance are divided into five groups, those who are significant at a Bonferroni corrected level of p < .05 (first and fifth rows), those who are significant at a non-Bonferroni-corrected level of p < .05 (second and fourth rows), and those who are not significant at a non-Bonferroni-corrected level of p < .05 (middle row). ‘Positive’ refers., arbitrarily, to examiners being more hawkish (i.e. giving lower overall scores), giving higher scores to male candidates, giving higher scores to White candidates, or giving higher scores to odd-numbered candidates. By chance alone one would expect 95% of candidates to be in the ‘non-significant’ group, with the remaining 5% of candidates distributed evenly between negative and positive effects.

The statistical analyses for each exam, PACES and nPACES, considered all of the individual judgements separately, comparing each and every judgement across the two examiners (and so there were many more comparisons for nPACES than PACES). Because the formats of the two examinations, and in particular the marking schemes, are very different, it is not straightforward to combine the two analyses into a single analysis. However, for present purposes the separate analyses of PACES and nPACES allow an assessment of the power and sensitivity of the method when there is a long series of examinations (26 in the case of PACES), and for a rather smaller set of examinations (6 in the case of nPACES). nPACES may in fact have more statistical power than PACES because each examiner makes more separate assessments of each candidate, on from four to seven different skills. In practical terms, it is also possible to compare the various indices of hawkishness, ethnic bias or sex bias in the two separate analyses, to assess their consistency.

Analyses were restricted to candidates for whom information on self-declared sex and ethnicity was available, these measures being missing in a few candidates, particularly those taking PACES when it was first introduced. Self-declared ethnicity and sex were known for all examiners.

Statistical analysis used IBM SPSS-20 for conventional analyses, and a special purpose program written in *Matlab* was used for the more detailed analyses. The *Matlab* program produced a file containing all the judgements made by all possible pairs of examiners. The program then systematically went through the file, one examiner at a time, comparing that examiner’s judgement with all of the judgements made by all of their co-examiners. The calculations are slightly different for the index of hawkishness, as opposed to the indices of ethnic bias, sex bias and odd number bias.

### Ethics

The work described in this paper was primarily ‘service evaluation’ and therefore was exempt from requiring permission from the UCL Research Ethics Committee (see http://ethics.grad.ucl.ac.uk/exemptions.php, section f). Statistical analyses were all carried out on data which were anonymised, using only code numbers to identify individuals, and were only de-anonymised on a ‘need-to-know’ basis for service needs.

### Hawkishness

For the examiner of interest, E, let there be *n* marks overall, which are called *m*_i_, *i* = 1,*n*. Each *m*_i_ is paired with a second mark, *s*_i_, *i* = 1,n, made by the co-examiners (C_j_, where there are *j* co-examiners, contributing 1 or 2 marks for PACES or 4 to 7 marks for nPACES). The difference between E’s marks and C’s marks, can be expressed as *d*_i_ = *s*_i_-*m*_i_. Notice that a hawk will tend to give lower marks, and hence *s*_i_-*m*_i_ will be positive for greater hawkishness. The *d*_i_ for examiner E can be tested to see whether overall there is systematic evidence for hawkishness by comparing mean(*d*_i_) with an expected value of zero, using a one-tailed *t*-test. A potential problem with the present method is that not all judgements in *m*_i_, *s*_i_ and *d*_i_ are necessarily statistically independent, being shared within a candidate or within a co-examiner. In principle more sophisticated analyses could be carried out using multilevel modelling for each examiner, but since our intention here was to produce a straightforward method which is easy to implement, we have not considered such models further. The fact that our test of ‘odd-number bias’ produces the expected results due to chance, suggests that any such effects are small enough to be of little consequence.

### Ethnic bias, sex bias and odd-number bias

In each case described here the basic analysis asks whether examiner E is treating candidates of type *u* differently from those of type *v*. The method is similar to that for hawkishness, except that the *d*_i_ scores can be divided into two non-overlapping sets, which we can call *u*_i_ and *v*_i_, *u*_i_ corresponding, say, to the *d*_i_ scores when the candidate was male, and *v*_i_ to the *d*_i_ scores when the candidate was female. If E treats the two groups equivalently, then *u*_i_ and *v*_i_ should have the same mean, a difference which can be tested using a standard two-sample *t*-test. If, say, E systematically scores males higher than females then *u*_i_ will be significantly higher than *v*_i_.

### Significance testing

The significance of the t-tests is calculated using a two-tailed test, because bias at either end of the scale (hawks or doves, male or females scored more highly) would have practical consequences, and because different individual examiners (who will themselves, for instance, be male or female, or white or non-white) may have different biases. A further consideration is that there is a large number of examiners, and hence some form of correction for alpha inflation due to multiple testing needs to be applied. If there are *N* examiners, then for normal scientific processes a typical Bonferroni correction would be to require that results are not significant at, say, the conventional p < .05 level but instead at the level of p < .05/*N*. In many ways the problem being addressed here, of identifying individuals whose behaviour is not merely anomalous, but could possibly have implications regarding probity or professional behaviour, is similar to that of identifying candidates whose pattern of responses in a multiple-choice examination, might well be construed as cheating [11]; in that case a higher criterion of significance such as 0.001 might be felt to be desirable (and it should also be Bonferroni corrected), although in the present paper, where the analyses are on an exploratory basis, we have used only the .05 level. Of course if there is a strong *a priori* reason for considering the behaviour of a particular examiner (such as in a case where there is independent evidence from a separate source), then the Bonferroni correction may not be appropriate.

## Results

Overall, 1790 examiners took part in the first 26 diets of PACES, examining an average of 135 candidates (range 13 – 912), and working with an average of 21 co-examiners (range = 1 – 136). The first six diets of nPACES were examined by 1498 examiners (1204 of whom had examined in the first 26 diets), who examined 65 candidates on average (range 12 – 625), and co-examined with an average of 8 co-examiners (range = 1 – 46%). The present analysis did not require a knowledge of the sex and ethnicity of the examiners, but in a recent census, 13.6% of UK examiners were female, 23% were non-White, and 24.2% were aged under 50 years. Examiners are therefore broadly representative of the Fellowship of the Royal Colleges of Physicians, which together include most consultant physicians in the UK, and of whom 18.3% were female, 23% were under 50 and 40% were non-White. Of the 17,442 candidates taking PACES or nPACES, 45.5% were female. Ethnicity was only known for 14,256 candidates, of whom 49.3% were non-White. Almost all candidates were aged under 50.

**Figure 1:**
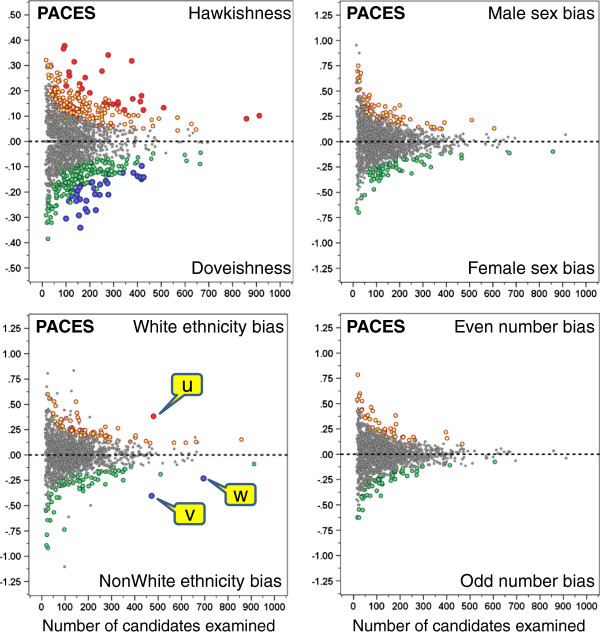
**The individual graphs show for PACES diets 1–26 the indices for hawkishness (top left), sex bias (top right), ethnic bias (lower left), and even-number bias (lower right).** Each point represents an individual examiner, plotted against the number of candidates examined, and with the significance indicated (grey, NS; orange and green p < .05 uncorrected; red and blue, p < .05 Bonferroni corrected).

**Figure 2:**
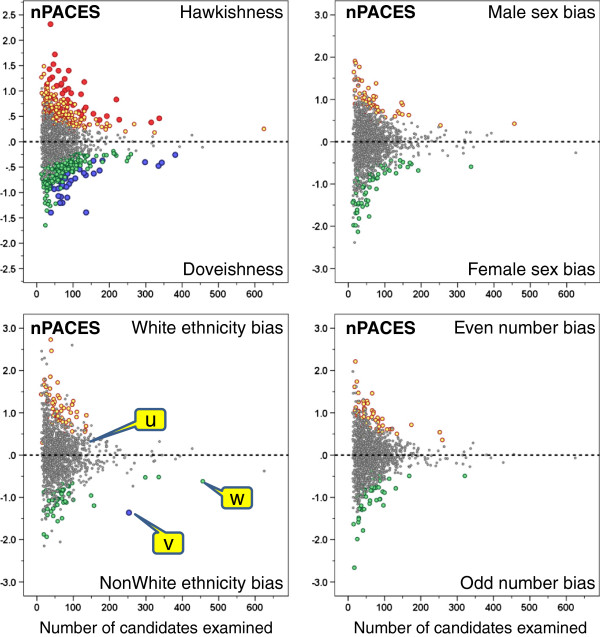
**The individual graphs show for the first six diet of nPACES (27 to 32) the indices for hawkishness (top left), sex bias (top right), ethnic bias (lower left), and even-number bias (lower right).** Each point represents an individual examiner, plotted against the number of candidates examined, and with the significance indicated (grey, NS; orange and green p < .05 uncorrected; red and blue, p < .05 Bonferroni corrected).

As described in the method section, for hawkishness, ethnic bias, sex bias and even-number bias, a *t*-test was calculated for each examiner, comparing all of their marks with the ‘basket’ of marks for the same candidates and stations from their co-examiners. Figures 1 and 2 show on the vertical axes the size of the various indices, a value of zero indicating either a typical extent of hawkishness, or the absence of effects due to sex, ethnicity or odd-even numbering. The scales of the vertical axes differ for the PACES and the nPACES exams, which is due to the marking categories being different in the two exams. The range of the sex bias, ethnic bias and even-number bias is different from the range of the hawk-dove effect, as difference scores are inherently more variable than the mean scores used for the hawk-dove effect. The statistical significance of an index of a particular size depends heavily on the total number of candidates examined, and that is shown on the horizontal axis. The significance of the points for individual examiners is shown by the colour of the circles, the small grey circles indicating p > .05. Coloured spots indicate P < .05 without any correction for repeated significance testing, and it would be expected that by chance alone, 5% of examiners would be in that category. The large red and blue dots indicate points that are significant at the 5% level of significance after a Bonferroni correction has been applied to correct for repeated significance testing. It should also be noted in Figures 1 and 2, particularly for sex and ethnic bias, that occasional points are non-significant (grey) despite being surrounded by coloured points, and hence one might have expected such points also to be significant. This effect arises because the significance of an effect also depends on the particular mix of male and female or white and non-white candidates that an examiner has seen, the *t*-test being most powerful when 50% of candidates are in each category.

Table 1 summarises the numbers of examiners in each of the significance categories for the two sets of exams, and the four different indices of examiner behaviour. It is clear that some examiners are significantly more hawkish or doveish than their co-examiners, as has been found previously [3]. Hawkishness and doveishness are found in relatively equal proportions and in both forms of the examination.

The odd-even numbering analysis finds that just over 5% of examiners showed (uncorrected)_significant levels of hawkishness (6.2% for PACES and 6.8% for nPACES, 95% confidence intervals using 1000 bootstrap replications, 4.7%-7.6% and 5.3%-8.2%), which is much as would be expected for what should be an entirely construct-irrelevant effect. Overall, it can probably be concluded that the method gives satisfactory results for what in effect is a random characteristic of candidates.

For sex bias, there is a slight excess of individuals outside the uncorrected 95% range (PACES: 8.5%; nPACES: 7.9%), although the proportions with positive (favouring male) and negative (favouring female) bias are approximately the same, indicating no overall bias in favour of one sex or the other (and it should be remembered that the majority of examiners are male). The results for ethnic bias show broadly similar results, again with a slight excess outside the uncorrected expected 95% range (PACES: 9.6%; nPACES 7.8%), and again with positive (favouring white) and negative (favouring non-white) biases being about equally distributed (and once again it should be remembered that a majority of examiners are white). For sex bias none of the individual examiners reach significance on the Bonferroni corrected criterion. The ethnic bias data in Figure 1 show that for PACES there were three examiners, labelled *u*, *v* and *w*, who were significant on the Bonferroni corrected criterion with p < .05. One examiner (*u*) is a white examiner in favour of white candidates and two examiners (*v* and *w*) are non-white examiners in favour of non-white candidates; *w* would not however reach significance on a stricter p < .001 criterion. For nPACES (Figure 2) only one examiner, *v*, reached the p < .05 Bonferroni-corrected criterion, and this examiner was also significant with p < .001 Bonferroni-corrected. Examiner *u* was non-significant on nPACES, and examiner *w* only reached an uncorrected p < .05 criterion. Only one examiner (*v*) reached the Bonferroni-corrected p < .05 significance level in both PACES and nPACES. This examiner was non-White and appeared to be systematically awarding relatively higher marks to non-White candidates.

### Correlations between PACES and nPACES

For many of the examiners there were two separate estimates of hawkishness, ethnic bias, sex bias and odd-even bias, one from PACES and the other from nPACES, and these two estimates can be correlated to find an estimate of the stability (reliability) of these indices. Hawkishness was calculated twice in 1204 examiners, and the correlation was 0.402 (p < .001), indicating strong stability. For sex bias the correlation in 1203 examiners was 0.091 (p = .002), and for ethnic bias the correlation for 1070 examiners was .145 (p < .001), indicating some stability, but a lot of variation. Finally, for the odd-number bias the correlation was 0.007 (p = .817), a result that is as might be expected given the way in which the indices were calculated. In interpreting all of these correlations it should be remembered that the indices are calculated on the basis of different numbers of judgements, and hence differ in their reliability, which may reduce the overall correlation of the two indices for PACES and nPACES.

### Correlations between bias indices

The extent of sex bias and ethnic bias showed a significant negative correlation in PACES (r = −.270, p < .001) and nPACES (r = −.265, p < .001), suggesting that examiners who rated white candidates more highly also rated female candidates more highly. Measures of hawkishness also correlated with sex bias (PACES, r = −.059, p = .012; nPACES r = −.161, p < .001), suggesting that more hawkish examiners rated male candidates more highly, but hawkishness only correlated with ethnic bias in nPACES (r = .302, p < .001) but not in PACES (r = .021, NS), the more hawkish nPACES examiners rating white candidates more highly.

### Simulating the situation where there is only a single examiner at each station

**Figure 3:**
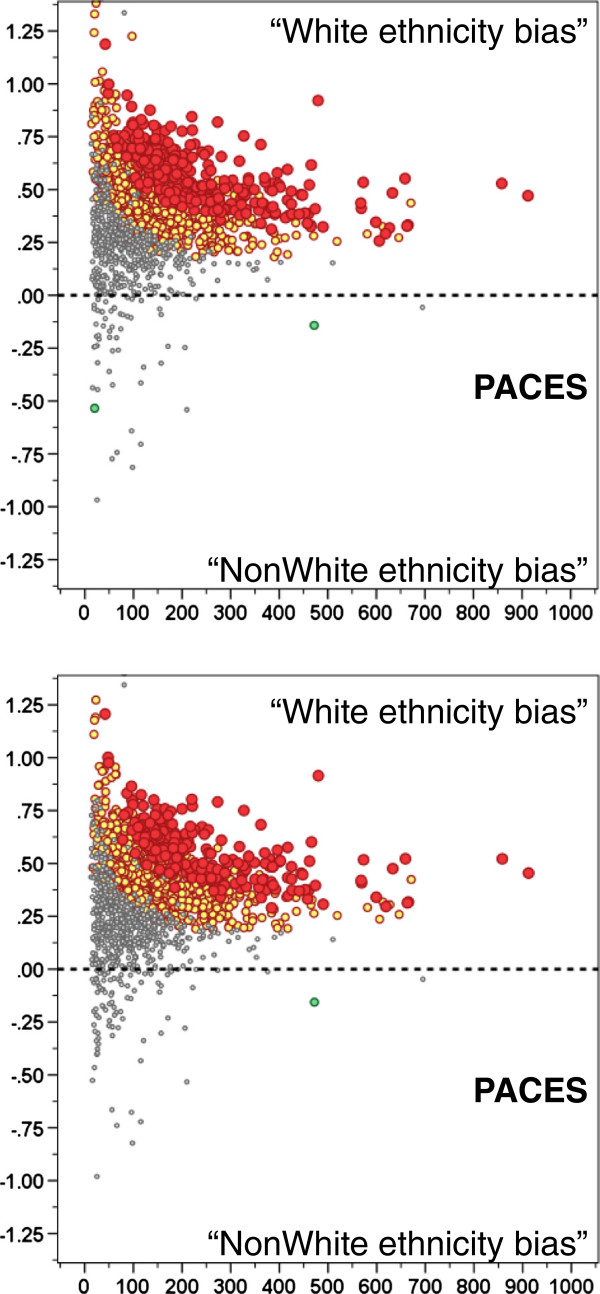
**Data from a simulated analysis of a single examiner at each station, based on the data for PACES diets 1–26.** Only ethnic bias is considered, for simplicity, with the top graph showing a simple comparison of White with non-white candidates, and the lower graph showing a more complex analysis in which individual marks are compared with a basket of marks awarded by other examiners at other stations. Each point represents an individual examiner, plotted against the number of candidates examined, and with the significance indicated (grey, NS; orange and green p < .05 uncorrected; red and blue, p < .05 Bonferroni corrected).

PACES and nPACES have two examiners at each encounter, whereas many other examinations have only a single examiner at each encounter. By considering only the marks awarded by just one of the PACES examiners, the situation of one examiner can be simulated and then compared with that of two examiners. For simplicity we here consider only the much larger PACES dataset in relation to ethnic bias. Two separate methods have been used:

1. *Simple comparison of marks given to White and non-white candidates*. A seemingly obvious method of assessing ethnic bias would be, for a single examiner, to find the mean of all the marks that have been given to White candidates and compare them to all the marks that have been given to non-White candidates, using a *t*-test to assess significance. Figure 3 (top) shows that with such a method a very large number of examiners apparently show bias towards white candidates. In the figure we have intentionally referred to “White ethnicity bias” in scare quotes, as the interpretation is wrong. In PACES, as we have shown before, non-White candidates, many of whom are international medical graduates, on aggregate perform less well than White candidates (a majority of whom are UK graduates). Whatever the underlying reasons for that, randomly chosen non-White candidates will on average perform less well than randomly chosen White candidates, and therefore for many examiners it will appear that they have a bias towards White candidates. The large preponderance of those apparently showing such a bias in Figure 3 (top) immediately throws doubt on such a suggestion. However had our analysis of PACES been based only on a single examiner, then very wrong conclusions could have been drawn. The comparison with the situation with two examiners, shown in Figure 1 (lower left), is striking.

2. *Comparison of a single examiner’s marks with those of other examiners on other stations*. A more sophisticated approach to the problem of single examiners might seem to involve using the marks of other examiners at other stations. We simulated this by firstly taking a series of marks of an individual examiner, E, on the candidates they had examined. One candidate at a time, we found the marks awarded to that candidate by all other examiners who were examining at other stations to E, calculated the mean of those other marks, and subtracted it from the other mark. The logic was that the basket of other marks awarded by the other examiners at other stations would provide an overall measure of the that candidate’s overall ability, and if an examiner was marking higher or lower than that basket then they may have biases of one form or another. The calculation was then similar to that in the previous example. Figure 3 (bottom) shows that the method still has a massive excess of examiners apparently showing a white ethnicity bias. The similarity to Figure 3 (top) and the difference from Figure 1 (lower left) suggest that an artefact is also at work here. The problem probably arises because marks on other stations are not a strong predictor of marks on the particular station on which the examiner examined. The result is that there is much content specificity, but since non-white candidates in general perform less well, they also perform less well on the specific content of the station on which that examiner happens to have examined.

## Discussion

In this paper we have presented a method for identifying behaviours in clinical examiners that can help to identify which are hawks and which doves, and also recognise behaviours consistent with ethnic or sex bias. We intentionally say, “consistent with”, because it is doubtful whether a simple statistical index alone can identify what are often complex behaviours underlying such possible biases. The indices we have examined are not strong enough to be able to identify such behaviours with certainty (and it is doubtful whether the method would be acceptable in a court of law). However the indices may form part of a broader set of evidence that might allow such identification, or be used to flag up behaviours which potentially are problematic for an examination. As it is, the indices certainly could provide evidence of biases which are statistically unlikely, and therefore require explanation, the logic, in many ways, being similar to that used in identifying candidates in multiple-choice examinations whose pattern of answering is ‘anomalous’, and requires explanation [11].

It seems that hawkishness is stable across time in our examiners, the correlation between PACES and nPACES being 0.402. Other work also suggests that hawkishness is found in most assessments, Myford [12] commenting that “the range of rater leniency/severity is typically about half that of rate performance” (p.408), making it an important source of variance. Although hawkishness is correlated across time, that alone does not mean hawkishness is a fixed trait in examiners, and it may be that training or self-awareness may modify hawkishness (although Eckes [13] has commented on how “rater severity differences are highly resistant to change … [so that] rater training … is, as a rule unlikely to meet with success”; p.72). Sex bias and ethnic bias also show correlations across PACES and nPACES, but the correlations are far smaller, with values of .091 and .145, suggesting that although there may be stability, relatively small amounts of variance on the two occasions are stable, the majority of the variance presumably being measurement error, in large part due to small Ns. Disattenuation of the correlations for unreliability is far from straightforward because of the large variation in sample sizes. Sex bias and ethnic bias are also correlated, and bias in favour of males is also correlated with hawkishness, suggesting that there may be underlying traits which are more general than just the characteristics described here.

The MRCP(UK) PACES and nPACES examinations are relatively unusual in having two separate examiners at each station. There are many reasons for that strategy having been adopted, but one result is that it is easy to carry out the analyses described here. Many exams have followed standard psychometric advice based on generalizability analyses and instead chosen to have more stations with only a single examiner. That strategy may have benefits in terms of increasing the reliability/generalizability of the assessment provided, as long as it is the case, as statisticians say, “all other things are remaining constant”. In social psychological terms, though, it is not clear that all other things do remain constant. Human beings often behave differently in the presence of other people, as opposed to when they think they are unobserved [14], both actual and implied ‘social presence’ altering behaviour [14]. Examiners might well be similar, the presence of another examiner concentrating the minds of each examiner, and resulting both in more reliable assessments and, perhaps also, less biased assessments. Certainly the rate of possible bias we have found in this large set of examiners is very small, with only one examiner out of over 2000 in total, having evidence consistent with an unusual amount of ethnic bias (and that bias is in favour of non-white rather than white candidates), and no examiners showing evidence of sex bias. Whether those rates would be the same were there to be no direct comparative group in the co-examiner is difficult to be certain about, but our intuition is that it would not be, and may be higher.

For the many clinical examinations with only a single examiner at a station it is less easy to assess examiner bias. Candidates may well see a range of examiners, but those examiners will be assessing on stations that vary in content and difficulty, both of which will add substantial variance, making it much harder, and therefore less powerful, for examiner bias to be detected with any confidence. Our data allow us to simulate the situation with a single examiner by simply considering the single mark of an examiner, either against their own marks for other candidates, or a basket of marks given to a candidate by other examiners. Figure 3 shows that neither method works well, seeming to identify large numbers of examiners as showing ethnic bias, mainly because the marks of the single examiner are confounded with the overall performance of the group of candidates as a whole. It should also be remembered that in this simulation it is indeed the case that “all other things do remain similar”, the marks being awarded in the presence of a second examiner, but analysed as if there was a single examiner. If the presence of a second examiner actually changes the behaviour of examiners then the effects would likely be larger still. It is not at all clear how statistical solutions can be developed to the problem of identifying examiner biases with only a single examiner at each station. A non-statistical possibility may be to have full video-recording, with other examiners subsequently reviewing cases and re-scoring them. Of course that still requires extensive examiner time, and in the final analysis would presumably be equivalent in examiner time to having two examiners at a station in the first place. An alternative strategy might be only to review video-tapes of examiners whom a preliminary analysis has suggested might be marginal in terms of their indices of bias, although even how that would work is not clear. It is clear that having two examiners, whatever the cost, makes a substantial contribution to exam probity, and to the ability to demonstrate probity.

The analysis of PACES was based on 27 diets and an average of 21 co-examiners, whereas the nPACES analysis had only 6 diets and an average of 8 co-examiners. Nevertheless the nPACES analysis confirmed one examiner as having a highly significant ethnic bias, and the individual sex bias and ethnic bias indices correlated significantly with those calculated for the same examiners who had examined in PACES. The implication is that the method is relatively sensitive even with smallish numbers of diets (although examiners do make more judgements for each candidate in nPACES than they do in PACES). Probably the most important feature is the number of diets, not candidates or marks awarded, since there have to be sufficient other examiners in the ‘basket’ for the comparison to be successful, although having more marks (as in nPACES) probably does increase the statistical power. How small the number of diets could be is far from clear at present, and it seems unlikely that it could be much lower than six (and a thought-experiment suggests that the method would inevitably fail with just a single diet, where each examiner will typically examine with only one or two other examiners, and most baskets will contain only one or two co-examiners).

The present analysis was prompted by a desire to analyse the possible presence of ethnic or sexual bias in clinical examiners in general, and to assess the potential for such analysis to be used to support the investigation of individual examiners who may be alleged to have displayed such bias by individual candidates. The method we describe found one examiner in an entire examiner pool of over 2000 examiners with possible ethnic bias and we believe that similar analyses could reasonably be used to support the investigation of individual allegations of bias if necessary in the future.

## Conclusions

Examiner bias is a potential risk in any examination. Although techniques for assessing overall tendencies to be a hawk or a dove have been described previously, here we describe a method for identifying examiners who show specific biases towards individuals with particular characteristics, such as those defined by sex or ethnicity. The method works effectively in an examination where there are two examiners per station.

## Abbreviations

MRCP(UK): Membership of the Royal Colleges of Physicians of the United Kingdom; nPACES: New Practical Assessment of Clinical Examination Skills; PACES: Practical Assessment of Clinical Examination Skills.

## Competing interests

The authors declare that they have no competing interests.

## Authors’ contributions

The idea for the present analysis was developed jointly by ICM and ATE. ICM carried out most of the statistical analyses and wrote the first draft of the paper, and JD and ATE worked on later drafts of the paper. All authors approved the final draft of the paper.

## Authors’ information

All three authors are involved with the MRCP(UK) examination, JD as Medical Director, ATE as Chair of the Clinical Examination Board, and ICM as Educational Adviser.

## Pre-publication history

The pre-publication history for this paper can be accessed here:

http://www.biomedcentral.com/1472-6920/13/103/prepub
